# The Potential of Triterpenoids from Loquat Leaves (*Eriobotrya japonica*) for Prevention and Treatment of Skin Disorder

**DOI:** 10.3390/ijms18051030

**Published:** 2017-05-11

**Authors:** Hui Tan, Tamrakar Sonam, Kuniyoshi Shimizu

**Affiliations:** Department of Agro-environmental Sciences, Faculty of Agriculture, Kyushu University, 6-10-1 Hakozaki, Higashi-ku, Fukuoka 812-8581, Japan; thth229@gmail.com (H.T.); tamrakar.snm@gmail.com (T.S.)

**Keywords:** *Eriobotrya japonica*, loquat leaves, triterpenoids, skin disorder, anti-melanogenesis, anti-acne, anti-allergy, anti-aging

## Abstract

The leaves of loquat (*Eriobotrya japonica*) possess high medicinal value and have been used as traditional medicines. However, there are no evidence-based studies on the skin-care effects of *E. japonica* leaves. To explore new biological activities of *E. japonica* leaves against skin disorder and to gain a better understanding of the chemical components associated with bioactivities, we evaluated 18 triterpenoids from *E. japonica* leaves on anti-melanogenesis, anti-acne, anti-allergy and anti-aging activities. Our results revealed that eight compounds showed anti-melanogenesis activity, of which ursolic acid (**1**) and maslinic acid (**7**) were the most potent with the similar selective index to that of arbutin. Structure–activity relationship and possible mechanism of active compounds were proposed. Twelve compounds exhibited anti-acne effect; ursolic acid (**1**), maslinic acid (**7**), corosolic acid (**8**) and euscaphic acid (**12**) showed highest activities against *P. acnes*. Four compounds displayed anti-allergy and anti-inflammatory activity; 3-epicorosolic acid (**9**) and euscaphic acid (**12**) showed marked activity against β-hexosaminidase release. Finally, ursolic acid (**1**), pomolic acid (**10**), colosolic acid (**8**) and its methylated derivative (**6**) exhibited the highest anti-aging activity by stimulating collagen and hyaluronic acid (HA) production. Our findings provide valuable evidence that *E. japonica* leaves have potential applications as ingredients of function foods or cosmetics for health benefits and a number of triterpenoids may play an important role in these bioactivities.

## 1. Introduction

The skin accounts for 15% of a human being’s total body weight, and it directly confronts the external environment serving as a primary defense system [1]. It plays a crucial role in not only preventing harmful substances, such as ultraviolet radiation, microbial pathogens and antigens from entering the body, but also in the maintenance of body homeostasis. Skin generally consists of a three-layer structure: the epidermis, the dermis and subcutaneous tissues. The epidermis is the outermost layer of the skin and keratinocytes are the most abundant cell type. Keratinocytes act via their capacity to produce UV-absorbing molecules, inflammatory mediators and antimicrobial peptides [2]. The dermis is the structure beneath the epidermis, its three primary structural components are collagen, elastin and glycosaminoglycans, which have been the subjects of the majority of anti-aging research [3].

Plant-derived natural, bioactive compounds have shown potential in beauty enhancement along with health benefits against disorders, such as photo-aging, inflammation, oxidation, aging, bacterial infections and so on [4]. These bioactive compounds continue to gain popularity based on their several advantages, which include often having fewer side effects, showing better patient tolerance, and being relatively less expensive and acceptable due to a long history of use compared to synthetic ingredients [5]. In the search for effective drug leads from herbs used against skin abnormalities, bioactive compounds such as aloin [6], curcumin [7], resveratrol [8], gallic acid [9], quercetin, genistein and catechins [10] have been found.

**Figure 1:**
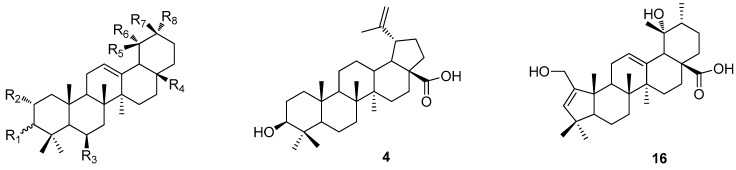
The structures of the 18 tested triterpenoids from *E. japonica* leaves.

Loquat (*Eriobotrya japonica*), a subtropical evergreen tree belonging to the Rosaceae family, is widely cultivated for its edible fruits. The leaves are well-known traditional Chinese medicine, used for the treatment of diabetic patients and for soothing effects on the digestive and respiratory systems. In Japan, loquat leaves are sold as a tasty herbal tea with health benefits. It has been reported that *E. japonica* leaves have great potential for the prevention of inflammation [11] and obesity [12]. In addition, loquat leaves may ameliorate Alzheimer’s disease, since *E. japonica* leaves protect against oxidative stress and cognitive deficits induced by the Aβ peptide [13]. Our previous studies showed that *E. japonica* have the potential to suppress ovariectomy-induced bone mineral density deterioration [14], and isolated triterpenoids as the major bioactive compounds suppress osteoclast differentiation [15]. As a powerful indigenous therapy, loquat leaves have also been used for skin-care application such as for sunburn, dermatitis, and skin aging. However, to the best of our knowledge, no study has investigated this bioactivity and desired chemical components from loquat leaves. Pentacyclic triterpenoids are present at their highest concentration in loquat leaves, and they have been reported to be related to various bioactivities. Therefore, in the present study we evaluated the potential involvement in skin-related bioactivity of a series of triterpenoids from *E. japonica* leaves (Figure 1), including anti-melanogenesis, anti-acne, anti-allergy, and anti-aging activity.

## 2. Results and Discussion

### 2.1. Anti-Melanogenesis Effect of Triterpenoids

Excessive ultraviolet (UV) radiation stimulates the production of melanin, which leads to various skin injuries, including inflammation, age spots, melasma and freckles [16]. We found previously that the methanol extract of *E. japonica* leaves exhibited significant and dose-dependent melanogenesis inhibition in B16 melanoma cells (Figure S1). In the present study, we focused on the potential anti-melanogenesis effect of 18 triterpenoids from *E. japonica* leaves. We tested various concentrations’ inhibitory effects on melanin synthesis and cell viability (Tables S1 and S2). The CC_50_ values for cell viability and the IC_50_ values against the melanogenesis of B16 cells are listed in Table 1. The relative effectiveness of the compounds for inhibiting melanogenesis compared to inducing cell death is defined as the selectivity index (S.I.). It is desirable to have a high selectivity index (S.I. > 1.0), which indicate the potential for selective melanogenesis inhibition. Our results demonstrated that eight of the compounds, namely ursolic acid (**1**), oleanolic acid (**2**), betulinic acid (**4**), methyl maslinate (**5**), methyl corosolate (**6**), maslinic acid (**7**), corosolic acid (**8**) and tormentic acid (**11**), showed melanin synthesis inhibitory activity with less cytotoxicity (S.I. > 1.0). Among them, ursolic acid (**1**) and maslinic acid (**7**) showed the highest S.I. values at 1.9, which is the same as the S.I. of arbutin, a skin-lightening ingredient commonly used in the cosmetic industry.

In addition, in our comparison of the bioactive chemical structures with those of inactive compounds, we found that the beta-form of the hydroxyl group at the R_1_ position may play an important role in the melanin synthesis inhibition activity, because there was a loss in this activity when the beta-form of the hydroxyl group was replaced by an alpha-form, (**9**) and (**12**), a ketone, (**13**)–(**15**), or another substituent group, (**17**) and (**18**). In addition, the carbonyl group at R_4_ position may partly contribute to the activity because uvaol (**3**), via the presence of a hydroxymethyl group at R_4_, loses its activity. The methyl esterification of the carboxyl group causes a slight loss of activity, since (**5**) and (**6**) showed weaker activity than (**7**) and (**8**).

**Figure 2:**
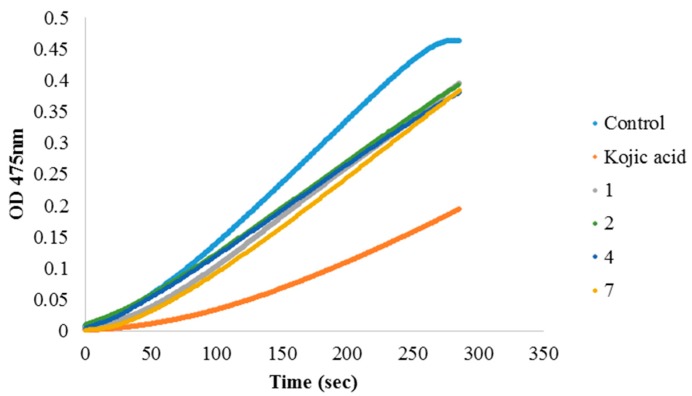
The effect of active compounds on tyrosinase activity (with respect to L-tyrosine). The inhibition curve of each compound was calculated under their maximum solubility of 330 μg/mL. Kojic acid is positive control.

Tyrosinase is the rate-limiting enzyme for controlling the production of melanin, it can catalyze the hydroxylation of tyrosine to 3,4-dihydroxyphenylalanine (DOPA) and the oxidation of DOPA to dopaquinone [17]. From dopaquinone, the melanin synthesis pathways diverge to produce either black-to-brown eumelanin or red-to-yellow pheomelanin. In the present study, we also evaluated a potential mechanism of the active compounds against melanin synthesis by performing a tyrosinase inhibitory assay. We use either L-tyrosine or L-DOPA as the substrates. The results revealed that compounds (**1**), (**2**), (**4**) and (**7**) showed moderate inhibitory activity on tyrosinase with L-tyrosine as substrate at relatively high concentrations, but no inhibitory effect in the case with L-DOPA as substrate (Figure 2). This result suggests that observed melanogenesis inhibition effect among eight active compounds, compounds (**1**), (**2**), (**4**) and (**7**) are at least partly contributed by tyrosinase inhibitory activity. Our study offers preliminary evidence for selecting potential candidates that may be used as skin-whitening agents and for further clearly exploring the mechanism of each compound, melanogenesis-related enzymes including melanocortin type 1 receptor (MC1R), tyrosinase-related proteins 1 and 2 (TRP1 and TRP2), and protein kinase regulators of melanogenesis-related signaling pathways (MAPK, PKA signaling pathways) need to be further discussed.

### 2.2. Anti-Bacterial Activity of Triterpenoids

Propionibacterium acnes (*P. acnes*) is an anaerobic gram-positive bacterium located primarily in the pilosebaceous unit of the skin. Together with the sebaceous gland, it plays an important role in the development of acne [18]. In our study, the antibacterial activity of triterpenoids against *P. acnes* was evaluated. Among these triterpenoids, 12 compounds, i.e., ursolic acid (**1**), oleanolic acid (**2**), methyl corosolate (**6**), maslinic acid (**7**), corosolic acid (**8**), pomolic acid (**10**), tormentic acid (**11**), euscaphic acid (**12**), 6β,19-dihydroxyurs-12-en-3-oxo-28-oic acid (**14**), hyptadienic acid (**16**), 3-*O*-*trans*-*p*-coumaroyltormentic acid (**17**) and 3-*O-cis-p*-coumaroyltormentic acid (**18**), were active against *P. acnes* at their maximum solubility. The minimum inhibitory concentration (MIC) and minimum bactericidal concentration (MBC) values of the active compounds are shown in Table 2. The results showed that ursolic acid (**1**), maslinic acid (**7**), corosolic acid (**8**) and euscaphic acid (**12**) showed the highest activities against *P. acnes* with an MIC value of 50 μg/mL and MBC values ranging from 50 to 150 μg/mL.

Although the triterpenoids showed low hydrophilicity with logP values ranging from 5 to 8, they also exhibited high levels of antibacterial activity in the present study. However, the exact mode of action of these triterpenes on bacteria, especially against *P. acnes*, is still unknown. Antibiotic therapy (such as that with tetracyclines, erythromycin and clindamycin) directed against *P. acnes* has been a mainstay of acne treatment for many years [19], but the drug resistance associated with these antibiotics is an increasing concern [20]. Our present findings may contribute to the development of new natural cosmetic products that can be used to prevent acne, and offer an alternative plant-derived antibacterial compounds against the growth of *P. acnes*.

### 2.3. Anti-Allergic and Anti-Inflammatory Effects of Triterpenoids

Allergy and inflammation can manifest as hyper-responsiveness to allergenic environmental substances in various target organs of the body (the skin, nose, lungs, gastrointestinal tract, etc.) [21]. In the case of skin as the primary interface between the body and the environment, inappropriate or misdirected immune activity results in inflammations such as skin rash, itching or eczema [22]. Allergic reactions are triggered when allergens cross-link preformed immunoglobulin E (IgE) bound to the high-affinity receptor (FcεRI) on mast cells [23]. The mast cells then degranulate and release vasoactive amines (mainly histamine), mediators (nitric oxide, prostaglandins and cysteinyl leukotrienes), chemokines and other cytokines [24].

Here we examined the anti-allergic effects by checking the release of β-hexosaminidase from IgE-stimulated RBL-2H3 mast cells. First, we selected several working concentrations for all tested compounds and the result demonstrated that four triterpenoids exhibited significant inhibitory activity at 25 μg/mL (*p* < 0.01; 25 μg/mL is maximum solubility) as shown in Table S3. Three triterpenoids in particular—ursolic acid (**1**, inhibitory rate 72.5%), 3-epicorosolic acid (**9**, inhibitory rate 54.4%) and euscaphic acid (**12**, inhibitory rate 74.5%)—significantly suppressed the release of β-hexosaminidase at a non-cytotoxic concentration. Maslinic acid (**7**) showed a suppression rate of 66% on β-hexosaminidase with 84.4% cell viability.

We also determined the IC_50_ values against the anti-allergy and anti-inflammatory activity of active compounds. As shown in Table 3, compounds (**1**), (**7**), (**9**) and (**12**) possessed inhibitory activity against β-hexosaminidase release with the IC_50_ value of 39.5, 22.86, 14.2 and 15.8 μM, respectively.

Our results are consistent with previous studies [25,26] showing that compounds (**1**), (**7**), (**9**) and (**12**) have significant inhibitory activity against inflammation induced by 12-*O*-tetradecanoylphorbol-13-acetate (TPA) in mice and TPA-induced Epstein–Barr virus early antigen activation. In the present study, we reveal a probable alternative mechanism to account for the anti-allergy and anti-inflammatory activity of the active compounds.

### 2.4. Skin Type I Collagen Production-Promoting Activity of Triterpenoids

Skin aging is characterized by atrophy of the dermal–epidermal junction with a decrease in mechanical tension and loss of elasticity, due mainly to marked reduction of the dermal extracellular matrix, including collagen, elastin, and hyaluronic acid [27,28]. Collagen secreted by normal human dermal fibroblasts (NHDFs) plays important roles in cell–cell adhesion, cell proliferation and cell differentiation, and thus the functional properties of the skin depends on the quality and condition of the collagen present in the dermis [29].

**Figure 3:**
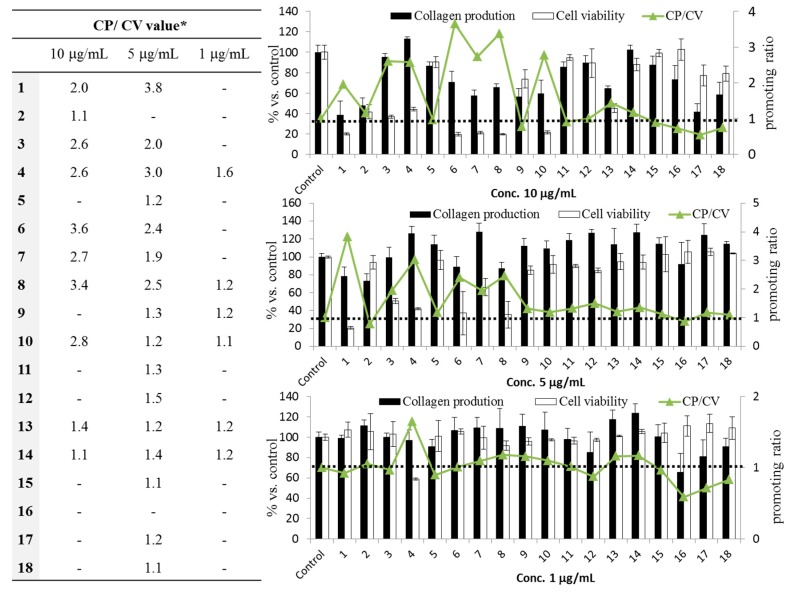
Collagen production (CP)—promoting effects of triterpenoids from leaves of *E. japonica* leaves. Data are the means ± SD of three independent tests, and all data were calculated as the percentage compared with the control value. Ascorbic acid (100 μM) was used as a positive control. * The promoting ratio (or CP/CV value) represents the mean of collagen production (%)/the mean of cell viability (CV) (%).

In our method, both the collagen production activity (CP) and cell viability (CV) were calculated individually at several concentrations for each compound. We then determined the CP/CV ratio (value > 1.0), which represents the “ratio of the mean of collagen production (%) to the mean of cell viability (%)” as an index of the stimulating activity of collagen production per cell. As shown in Figure 3, our results demonstrated that some triterpenoids can significantly enhance the dermal fibroblast collagen production per cell. Of the 18 test triterpenoids, ten induced type I collagen production at a concentration of 10 μg/mL, with methyl corosolate (**6**) and corosolic acid (**8**) showing especially potent stimulation of up to 3.4- and 3.6-fold that of the control, and the rest of the compounds showed a moderate promoting effect. At 5 μg/mL, 16 compounds triggered increased collagen production, with ursolic acid (**1**) and betulinic acid (**4**) increasing the collagen production up to 3.8- and 3.0-fold over the control level, respectively. Six compounds showed collagen-promoting activity at low concentrations. Concentrations higher than 10 μg/mL are not shown because of cytotoxicity.

Our results may contribute to the development of new cosmeceuticals and formulas from natural resources that can facilitate both the repair of skin wrinkles and fight against skin aging [30].

### 2.5. Stimulating Effect of Triterpenoids on Hyaluronic Acid Synthesis

The loss of the skin’s high water content is one of the hallmarks of skin aging. The key molecule involved in skin moisture is hyaluronic acid, which is a high molecular weight glycosaminoglycan with a unique capacity to retain water [31]. Increasing amount of hyaluronic acid is also important for the wound healing process [32].

**Figure 4:**
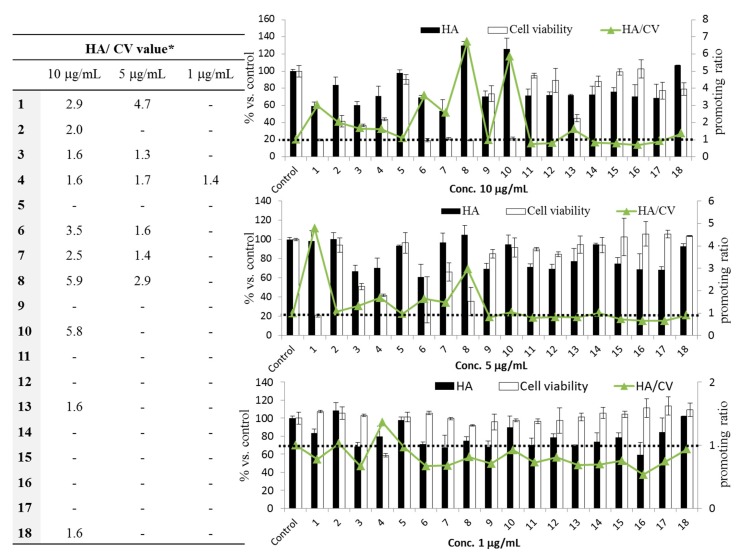
Stimulatory effects of triterpenoids from *E. japonica* leaves on hyaluronic acid (HA) production. Data are the means ± SD of three independent tests, and all data were calculated as percentages compared with the control values. Ascorbic acid (100 μM) was used as a positive control. * The promoting ratio (or HA/CV value) represents the mean of hyaluronic acid (%)/the mean of cell viability (%).

We therefore evaluated the potential effect of triterpenoids on hyaluronic acid production (Figure 4). At 10 μg/mL, ten compounds exhibited a potent stimulatory effect on hyaluronic acid production in human dermal fibroblasts, with the compounds corosolic acid (**8**) and pomolic acid (**10**) showing the strongest hyaluronic acid stimulating activity of 5.9- and 5.8-fold that of the control, respectively; methylation derivative (**6**) also showed a high stimulating effect. At 5 μg/mL, six compounds stimulated hyaluronic acid production, and it is worth noting that ursolic acid (**1**) achieved a 4.7-fold increase in hyaluronic acid (HA) production. At a low concentration (1 μg/mL), however, only betulinic acid (**4**) had a promoting effect. Concentrations higher than 10 μg/mL are not shown because of cytotoxicity.

Our present findings did not reveal a direct reason for the accumulation of collagen and hyaluronic acid by triterpenoids, but a pair of studies have shown that triterpenes extracted from the medicinal herb *Centella asiatica* induced changes in hyaladherin TNF alpha induced protein 6 (TNFAIP6) and cytokine expression, and both of these changes were expected to lower the rate of proteolysis in the extracellular matrix and support accumulation collagen, the triterpenes did not directly affect the expression levels of collagen gene [33,34]. It seems that the stimulation of hyaluronic acid synthesis by triterpenoids could also account for the inhibitory effect on hyaluronidase, an enzyme that degrades hyaluronic acid by cleaving the *N*-acetylglucosamidic bonds of hyaluronic acid via a β-elimination process to produce hyaluronic acid oligosaccharides [35].

## 3. Materials and Methods

### 3.1. Chemicals

Triterpenoids isolated from *E. japonica* leaves for biological activities were obtained as follow: Ursolic acid (**1**) was obtained from Wako Pure Chemical Industries (Osaka, Japan); Oleanolic acid (**2**) was from Cayman Chemicals (Ann Arbor, MI, USA); Uvaol (**3**) was obtained from Extrasynthese (Genay Cedex, France); Betulinic acid (**4**) was obtained from Focus Biomolecules (Plymouth Meeting, PA, USA); Maslinic acid (**7**) was obtained from Cayman Chemicals; Pomolic acid (**10**) was obtained from Quality Phytochemicals (East Brunswick, NJ, USA); Corosolic acid (**8**), 3-epicorosolic acid (**9**), tormentic acid (**11**), euscaphic acid (**12**), 3-oxours-12-en-28-oic acid (**13**), 6β,19-dihydroxyurs-12-en-3-oxo-28-oic acid (**14**), hyptadienic acid (**16**), 3-*O-trans-p*-coumaroyl tormentic acid (**17**) and 3-*O-cis-p*-coumaroyl tormentic acid (**18**) were obtained from Chemfaces (Wuhan, China). Methyl maslinate (**5**), methyl corosolate (**6**) and 2α, 19α-dihydroxy-3-oxo-12-ursen-28-oic acid (**15**) were previously isolated from *E. japonica* leaves. The purity of all tested compounds was above 95% as verified by analytical high-performance liquid chromatography (HPLC).

### 3.2. Anti-Melanogenesis Assay

This assay was performed as described by Arung et al. [17]. The mouse melanoma cell line B16 was obtained from the Riken Bioresource Center Cell Bank (Tsukuba, Japan). The B16 melanoma cells were maintained in Eagle’s Minimal Essential Medium (EMEM, Nissui Chemical, Tokyo) supplemented with 10% (*v*/*v*) fetal bovine serum (FBS; Gibco, Carlsbad, CA, USA) and 0.09 mg/mL theophylline (Sigma-Aldrich, St. Louis, MO, USA). The cells were placed in 24-well plastic culture plates at a density of 1 × 10^5^ cells/well and incubated for 24 h in media prior to being treated with the samples. After 24 h, test samples were dissolved in DMSO (<0.2% per well) and sample solution were added to each well. Arbutin (Tokyo Kasei Kogyo, Tokyo) was used as a positive control. The cells were incubated for an additional 48 h, and then the medium was replaced with fresh medium containing each sample again. After 24 h, the melanin content and the cell viability were measured using a microplate reader (BioTek-ELX800, BioTek Instruments, Winooski, VT, USA) at 405 nm and 570 nm, respectively. Cell viability was measured using the MTT (3-(4,5-dimethylthiazol-2-yl)-2,5-diphenyltetrazolium bromide) method.

### 3.3. Tyrosinase Inhibition Assay Assay

Tyrosinase inhibition activity was evaluated by using L-tyrosine and L-DOPA as substrates, as described [36]. Briefly, 333 μL of 2.5 mM L-tyrosine or L-DOPA solution (Sigma-Aldrich) was mixed with 600 μL of 0.1 M phosphate buffer (pH 6.8) and incubated at 25 °C. Then, 33 μL of the sample solution (dissolved in DMSO in various concentrations) and 33 μL of 1380 units/mL mushroom tyrosinase (Sigma-Aldrich) were added to the mixture. The optical density was measured at 475 nm up to the appropriate time. Kojic acid (Sigma-Aldrich) was used as the positive control; the final concentration is 3.3 μg/mL.

### 3.4. Anti-Bacterial Assay

The antibacterial assay was based mostly on the methods described by Tanaka et al. with slight modification [37]. *Propionibacterium acne* (NBRC 107605) was used for the antibacterial assay. A single colony of the test strain was taken and 5 mL of Gifu anaerobic medium (GAM) broth (Nissui, Tokyo) was added to it. This culture was incubated at 37 °C for 30 h. It was then added to the bacterial suspension to prepare a bacterial concentration at 10^5^ CFU/mL. The bacterial solution was used for the subsequent antibacterial assay. Each compound was dissolved in DMSO, and benzalkonium chloride (Wako) was used as a positive control. The plate was incubated at 37 °C for 30 h. Finally, bacterial growth was measured by a microplate reader at 630 nm. The MIC and MBC were evaluated by the standard broth microdilution method.

### 3.5. Anti-Allergic and Anti-Inflammatory Assay

The anti-allergy assay was based mostly on the methods described by Zhu et al. [38] and Yun et al. with slight modification [39]. Rat basophilic leukemia (RBL-2H3) cells were obtained from the Riken Bio-resource Center Cell Bank (Tsukuba, Japan). RBL-2H3 cells were incubated in EMEM medium containing 10% FBS and 1% antibiotics–antimycotics at 37 °C. The cells were seeded on a 96-well plate (2 × 10^5^ cells/well). After 24 h of incubation, the medium was supplemented with 0.5 μg/mL anti-DNP (dinitrophenol) mouse immunoglobulin E (IgE) (Sigma-Aldrich). After an additional 24 h of incubation, the medium was replaced with 100 μL of Tyrode buffer. The sample dissolved in DMSO (<0.5% per well) was added, and the cell plate was incubated again for 30 min, then replaced with 100 μL of tyrode buffer containing 50 μg/mL DNP-bovine serum albumin (BSA) (Invitrogen, Carlsbad, CA, USA). After 1 h of incubation, 50 μL of the supernatant was collected from each well and mixed with 50 μL of substrate solution (*p*-nitrophenyl-*N*-acetyl-β-glucosaminide, 2 mM; Sigma-Aldrich) in a new 96-well plate. The mixture was incubated at room temperature on a shaker for 3 h. Finally, 100 μL of stop solution was added, and the absorbance was read at 405 nm. A parallel MTT experiment was performed to measure the cell viability. The MTT experiment was done exactly as described [40].

### 3.6. Skin Collagen Synthesis Promoting Assay

The assay for measuring the promotion of skin collagen synthesis was described by Kishikawa et al. [41]. Adult NHDF cells (Lonza, Tokyo) were routinely maintained in 10% FBS and 1% antibiotic-antimycotic (Invitrogen) in Dulbecco’s modified Eagle’s medium (DMEM, Wako). After growing to confluence, cells at a density of 5 × 10^3^ cells/well were seeded on 96-well plates for 24 h before the treatment. The medium was replaced with a mixture of 1 μL of sample solution (DMSO < 0.5% per well) and 99 μL of DMEM supplemented with 0.5% FBS. After 72 h of cultivation, the amount of collagen in the medium was measured using a human type I collagen enzyme-linked immunosorbent assay (ELISA) kit (ACEL, Kanagawa, Japan). The amount of collagen production was calculated using a standard curve prepared by measurement by the same ELISA. The cells remaining in the 96-well plate were subjected to an MTT assay. Ascorbic acid (100 μM, Wako) was used as a positive control.

### 3.7. Hyaluronic Acid Production Assay

NHDF-Ad cells were seeded on 96-well plates (5 × 10^3^ cells/well) for 24 h before the treatment. The medium was replaced with a mixture of 1 μL of sample solution (DMSO < 0.5% per well) and 99 μL of DMEM supplemented with 0.5% FBS. After 72 h cultivation, the amount of hyaluronic acid in the medium was measured using a QnE Hyaluronic acid ELISA kit (Biotech Trading Partners, Encinitas, CA, USA). The amount of hyaluronic acid production was quantitatively calculated from the standard curve. Cell viability was measured by the MTT method, and ascorbic acid was used as a positive control.

### 3.8. Statistical Analysis

All values from three independently repeated experiments are presented as the mean ± standard deviation. The significance of differences between means for two groups was determined by Student’s *t*-test. Differences were considered significant at * *p* < 0.05 and ** *p* < 0.01. The IC_50_ and CC_50_ values were calculated from the corresponding dose–response curves in at least four concentrations.

## 4. Conclusions

By establishing a complete skin-related bioactivity evaluation method and using previous studied secondary metabolites from the leaves of *E. japonica*, we have obtained a better understanding of the multiple functions of *E. japonica* leaves, and provided an evidence-based study that *E. japonica* leaves can be used to prevent skin-related problems. It is clear that triterpenoids from *E. japonica* leaves exhibit powerful skin-protecting effects, such as the inhibition of melanogenesis, *P. acnes* growth and allergies, and the promotion effects for collagen/hyaluronic acid production. In contrast to the previously identified anti-melanogenesis, anti-acne, anti-allergy, and anti-aging triterpenoids, newly identified and more potent inhibitors were identified in our study.

In terms of anti-melanogenesis activity, we identified eight compounds that have melanin synthesis inhibitory activity. By means of a structure–activity relationship study, we deduced the functional groups that contribute to activity. The mechanism underlying four active compounds’ melanogenesis inhibition effect is partly associated with their tyrosinase-inhibitory activities. There has been very little reported research about *P. acne* growth inhibition by triterpenoids, and here we found twelve compounds that were active against *P. acnes* at their maximum solubility. These compounds could be considered an alternative source to combat antibiotics resistance. However, the mechanism of action of each active component remains unknown and further research of this chemical class is needed. With regards to anti-allergy activity, our present findings revealed an alternative mechanism of active triterpenoids from *E. japonica* leaves by suppressing β-hexosaminidase. In addition, treatment using triterpenoids from the leaves of *E. japonica* can efficiently restore collagen and hyaluronic acid synthesis, as we observed a 3- to 4-fold increase in the level of collagen content and a 5- to 6-fold increase in hyaluronic acid synthesis.

Triterpenes represent a promising class of multi-target agents. In this study, the compounds ursolic acid (**1**) and maslinic acid (**7**) showed multiple activities against different skin-related problems. The mechanism underlying their multi-targets is interesting. The synergistic effects of these triterpenoids should also be considered. The results of this study indicate that *E. japonica* leaves have great potential to be used as ingredients in function food for increasing nutritional value and in cosmetics for skin-care effect.

## Figures and Tables

**Table:** 

Compound	R_1_	R_2_	R_3_	R_4_	R_5_	R_6_	R_7_	R_8_
**1**	β–OH	H	H	COOH	Me	H	H	Me
**2**	β–OH	H	H	COOH	H	H	Me	Me
**3**	β–OH	H	H	CH_2_OH	Me	H	H	Me
**5**	β–OH	OH	H	COOCH_3_	H	H	Me	Me
**6**	β–OH	OH	H	COOCH_3_	Me	H	H	Me
**7**	β–OH	OH	H	COOH	H	H	Me	Me
**8**	β–OH	OH	H	COOH	Me	H	H	Me
**9**	α–OH	OH	H	COOH	Me	H	H	Me
**10**	β–OH	H	H	COOH	Me	OH	H	Me
**11**	β–OH	OH	H	COOH	Me	OH	H	Me
**12**	α–OH	OH	H	COOH	Me	OH	H	Me
**13**	=O	H	H	COOH	Me	H	H	Me
**14**	=O	H	OH	COOH	Me	OH	H	Me
**15**	=O	OH	H	COOH	Me	OH	H	Me
**17**	β–*trans-p*-coumaroyl	OH	H	COOH	Me	OH	H	Me
**18**	β–*cis-p*-coumaroyl	OH	H	COOH	Me	OH	H	Me

**Table 1:** Anti-melanogenesis effect of triterpenoids from *E. japonica* leaves.

No.	CC_50_^a^ (μM)	IC_50_^b^ (μM)	S.I ^c^	No.	CC_50_ (μM)	IC_50_ (μM)	S.I ^e^
**1**	9.4	4.8	1.9	**10**	10.5	14.0	0.8
**2**	39.9	26.8	1.5	**11**	23.3	18.5	1.3
**3**	>45.2	>45.2	n.d. ^d^	**12**	25.6	34.0	0.7
**4**	16.7	11.8	1.4	**13**	8.9	18.0	0.5
**5**	17.5	12.8	1.4	**14**	10.9	14.8	0.7
**6**	18.7	16.1	1.2	**15**	>42.3	>42.3	n.d.
**7**	29.3	18.5	1.9	**16**	16.5	21.6	0.8
**8**	25.8	18.7	1.4	**17**	24.0	>30.9	n.d.
**9**	>42.3	>42.3	n.d.	**18**	19.5	>30.9	n.d.

^a^ The CC_50_ value is defined as the cytotoxic concentration causing death in 50% of viable cells, and is calculated from the corresponding dose–response curves (for the individual concentrations see Table S1); ^b^ The IC_50_ value is defined as the concentration required for 50% inhibition of melanin synthesis, and is calculated from the corresponding dose–response curves (for the individual concentrations see Table S2); ^c^ The selectivity index (S.I.) was calculated as the ratio of the concentration of the compound that is required to reduce the cell viability to 50% to the concentration of the compound needed to inhibit melanogenesis to 50% of the control value (i.e., the CC50 value/IC50 value); ^d^ n.d.: non-detectable; ^e^ Arbutin was used as a positive control, its S.I was calculated as 1.9.

**Table 2:** Anti-acne effect of triterpenoids from *E. japonica* leaves.

No.	Concentration μg/mL	Inhibition Rate %	MIC ^a^/MBC ^b^ μg/mL	No.	Concentration μg/mL	Inhibition ^e^ Rate %	MIC/MBC μg/mL
**1**	100	109.8 ± 0.5	50/100	**10**	100	84.4 ±1.2	n.d.
**2**	100	100.4 ± 0.8	100/200	**11**	100	102.8 ± 2.1	100/200
**3**	50	– ^c^	–	**12**	100	98.5 ± 1.3	50/50
**4**	100	–	–	**13**	100	–	–
**5**	100	–	–	**14**	100	87.6 ± 7.4	n.d.
**6**	100	77.9 ± 1.6	n.d. ^d^	**15**	100	–	–
**7**	100	104.1 ± 3.1	50/150	**16**	100	103.9 ± 3.1	100/200
**8**	100	105.9 ± 0.4	50/100	**17**	100	101.4 ± 2.1	100/200
**9**	100	–	–	**18**	100	103.5 ± 2.2	100/200

^a^ The minimum inhibitory concentration (MIC) is the lowest concentration of an anti-bacterial agent required to completely inhibit the growth of a particular bacterium; ^b^ The minimum bactericidal concentration (MBC) is the lowest concentration of an antibacterial agent required to kill the bacterium; – ^c^ : no inhibition; n.d. ^d^ : non-detectable; ^e^ Benzalkonium chloride was used as the positive control and showed 100% inhibition of bacterial growth at the conc. of 200 μg/mL.

**Table 3:** Anti-allergy and anti-inflammatory effects of triterpenoids from *E. japonica* leaves.

No.	IC_50_ (μM)	25 μg/mL	12.5 μg/mL	5 μg/mL	1 μg/mL
		Inhibition Rate (%)			
**1**	39.5	72.5 ± 3.0 **	37.6 ± 3.0 *	0.0 ± 11.5	n.d.
**7**	22.8	66.0 ± 2.6 *	58.2 ± 3.0 *	9.5 ± 6.6	n.d.
**9**	14.2	54.4 ± 4.9 *	50.7 ± 3.2 *	33.7 ± 6.9	8.9 ± 5.2
**12**	15.8	74.5 ± 1.3 **	67.0 ± 1.8 **	37.7 ± 3.9 *	10.4 ± 10.8

Quercetin was used as the positive control and showed the inhibition rate of β-hexosaminidase release of 72.5 ± 3.0% without cytotoxicity at 82 μM. n.d.: non-detectable. * *p* < 0.05 and ** *p* < 0.01.

## Supplementary Materials

Supplementary materials can be found at www.mdpi.com/1422-0067/18/5/1030/s1.

## Abbreviations

CV	Cell viability
CP	Collagen production activity
HA	Hyaluronic acid
MIC	Minimum inhibitory concentration
MBC	Minimum bactericidal concentration
S.I.	Selectivity index
