# Supplementary material for: The Potential of Triterpenoids from Loquat Leaves (Eriobotrya japonica) for Prevention and Treatment of Skin Disorder
Source: Int J Mol Sci. 2017 May 11;18(5):1030. doi: 10.3390/ijms18051030 (PMC5454942; doi:10.3390/ijms18051030)
Supplement: Supplementary file 1 [file ijms-18-01030-s001.pdf]

Supplementary material

The potential of triterpenoids from loquat leaves (*Eriobotrya japonica*) for prevention and treatment of skin disorder

Hui Tan [1], Tamrakar Sonam [1] and Kuniyoshi Shimizu [1,*]

[1] Department of Agro-environmental Sciences, Faculty of Agriculture, Kyushu University, 6-10-1 Hakozaki, Higashi-ku, Fukuoka, 812-8581, Japan; thth229@gmail.com (H.T.); tamrakar.snm@gmail.com (T.S.)
* Correspondence: shimizu@agr.kyushu-u.ac.jp; Tel.: +81 092 642 3002

Content:

Supplementary Figure S1. Anti-melanogenesis effect of methanol extract of *E. japonica* leaves……………………………………………………………………………………………..…………..I

Supplementary Table S1. The cell viability of triterpenoids on B16 melanoma cell in each individual concentrations……..…..II

Supplementary Table S2. Anti-melanogenesis effect of triterpenoids in each individual concentrations……………………………………………………………………………………..…III

Supplementary Table S3. Anti-allergy and anti-inflammatory effect of triterpenoids at 25 μg/ml…...IV

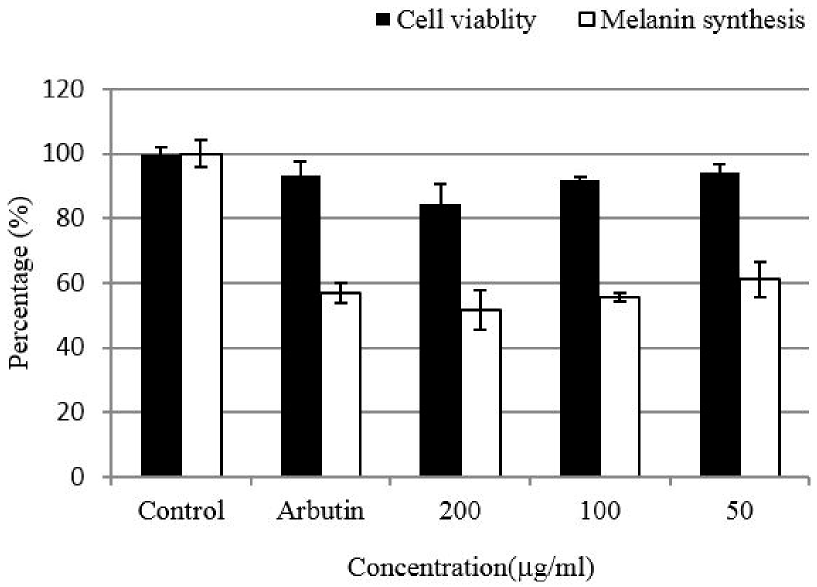

Figure S1. Anti-melanogenesis effect of methanol extract of *E. japonica* leaves. Arbutin (100 μg/ml) was used as a positive control.

Table S1. The cell viability of triterpenoids on B16 melanoma cell in each individual concentrations.

Compound[d]	CC_{50}[a] cell viability		20 μg/ml	10 μg/ml	5 μg/ml	2.5 μg/ml	1 μg/ml
	(μM)	(μg/ml)	Inhibition rate[b] (%)				
**1**	9.4	4.3	n.d.[c]	n.d.	54.8 ± 7.0**	31.2 ± 11.7*	0.0 ± 4.4
**2**	39.9	18.2	54.9 ± 2.3**	27.4 ± 7.0**	17.0 ± 6.2*	7.8 ± 4.1*	4.9 ± 4.6
**3**	> 45.2	>20.0	6.5± 7.4	10.8 ± 6.8	4.0 ± 7.6	n.d.	n.d.
**4**	16.7	7.6	68.1 ± 11.4**	58.4 ± 14.1**	35.9 ± 5.2**	20.8 ± 5.2**	1.2 ± 12.0
**5**	17.5	8.4	n.d.	75.9 ± 0.5**	12.1 ± 8.6	1.3 ± 5.2	4.7 ± 8.1
**6**	18.7	9.1	n.d.	59.4 ± 7.0**	15.4 ± 5.1**	10.5 ± 10.9*	4.5 ± 13.7
**7**	29.3	13.8	76.5± 3.0**	44.3 ± 6.4**	42.2 ± 4.0**	3.2 ± 4.8	2.5 ± 2.2
**8**	25.8	12.2	94.8± 0.3**	39.3 ± 4.7**	0.3 ± 4.0	0.0 ± 3.8	0.0 ± 3.5
**9**	> 42.3	>20.0	14.8 ± 4.1*	3.4 ± 4.1	0.0 ± 11.8	0.0 ± 10.3	n.d.
**10**	10.5	4.9	n.d.	68.4 ± 1.5**	50.9 ± 4.4**	0.0 ± 6.1	1.9 ± 5.4
**11**	23.3	11.4	85.8 ± 1.6**	52.4 ± 8.6*	8.7 ± 12.1	13.7 ± 10.7	12.0 ± 12.8
**12**	25.6	12.5	91.1 ± 1.0**	44.9 ± 2.7**	0.0 ± 6.6	0.0 ± 6.9	n.d.
**13**	8.9	4.0	n.d.	80.3 ± 3.0**	51.5 ± 3.7**	42.7 ± 2.1**	39.1 ± 2.6**
**14**	10.9	5.3	n.d.	69.1 ± 10.0**	44.1 ± 0.4**	1.2 ± 9.7	2.5 ± 8.3
**15**	> 42.3	>20.0	26.4 ± 1.4**	11.4 ± 3.9*	5.6 ± 4.6	n.d.	n.d.
**16**	16.5	7.8	n.d.	60.3 ± 6.5**	28.9 ± 3.2**	14.7 ± 4.2*	3.7 ± 3.8
**17**	24.0	15.6	61.0 ± 1.6**	41.1 ± 9.2**	10.7 ± 10.0	5.1 ± 6.0	0.0 ± 8.4
**18**	19.5	12.6	80.1 ± 9.5**	42.4 ± 14.9*	12.6 ± 8.4	6.6 ± 11.1	7.0 ± 8.8

[a]. CC_{50} value is defined as the concentration at 50% inhibition on cell viability, calculated from corresponding dose response curves;

[b]. Inhibition rate (%) = 100% − cell viability (%) of each tested compound;

[c]. n.d.: not determined;

[d]. Arbutin was used as positive control and showed CC_{50} value of 1820.2 μM.

Table S2. Anti-melanogenesis effect of triterpenoids in each individual concentrations.

Compound[d]	IC50 [a]melanin		20 μg/ml	10 μg/ml	5 μg/ml	2.5 μg/ml	1 μg/ml	0.5 μg/ml
	(μM)	(μg/ml)	Inhibition rate[b] (%)					
**1**	4.8	2.2	n.d.[c]	n.d	n.d.	58.5 ± 3.2**	24.0 ± 0.8**	4.2 ± 1.7
**2**	26.8	12.2	58.6 ± 2.3**	45.9 ± 1.5**	33.5 ± 6.0**	27.8 ± 4.8**	9.5± 4.6	n.d.
**3**	> 45.2	>20.0	14.6± 4.6**	14.2 ± 2.6**	0.0 ± 6.9	n.d.	n.d.	n.d.
**4**	11.8	5.3	n.d.	54.0 ± 3.3**	49.7 ± 3.1**	44.8 ± 1.4**	35.4 ± 0.5**	23.2 ± 1.4**
**5**	12.8	6.2	n.d.	68.2 ± 0.6**	44.8 ± 4.3**	1.6 ± 0.6	0.0 ± 4.4	n.d.
**6**	16.1	7.8	n.d.	63.5 ± 2.7**	32.7 ± 1.5**	2.8 ± 4.5	0.0 ± 1.9	n.d.
**7**	18.5	8.7	65.1± 0.4**	54.5 ± 3.0**	47.5 ± 0.4**	12.2 ± 1.9	2.8 ± 1.1	n.d.
**8**	18.7	8.8	n.d.	59.8 ± 1.9**	17.4 ± 5.5*	0.5 ± 4.1	3.4 ± 5.1	n.d.
**9**	> 42.3	>20.0	7.8± 0.9	n.d.	0.0 ± 5.3	1.0 ± 5.4	n.d.	n.d.
**10**	14.0	6.6	n.d.	55.8 ± 8.1**	43.9 ± 1.9**	28.4 ± 1.5**	3.2 ± 8.2	n.d.
**11**	18.5	9.0	73.4 ± 8.0**	51.3 ± 0.9**	34.8 ± 4.5**	11.3 ± 3.4	0.0± 6.1	n.d.
**12**	34.0	16.6	58.8 ± 0.0**	35.0 ± 3.6**	0.0 ± 3.6	0.0 ± 7.2	n.d.	n.d.
**13**	18.0	8.1	n.d.	52.9 ± 0.6**	45.5 ± 2.7**	39.7 ± 0.6**	41.0 ± 2.1	37.4 ± 1.1**
**14**	14.8	7.2	n.d.	67.3 ± 1.4**	39.5 ± 3.1**	15.8 ± 1.8*	6.9 ± 1.8	n.d.
**15**	> 42.3	>20.0	n.d.	24.2 ± 1.0**	n.d.	n.d.	n.d.	n.d.
**16**	21.6	10.1	n.d.	51.8 ± 5.0**	13.4 ± 5.5**	1.9 ± 3.4	0.1 ± 4.5	n.d.
**17**	> 30.9	>20.0	30.5 ± 6.0**	29.4 ± 6.8**	11.1 ± 0.6*	6.2 ± 1.7	2.8 ± 0.9	n.d.
**18**	> 30.9	>20.0	47.1 ± 6.0**	35.6 ± 1.1**	0.0 ± 3.8	0.0 ± 5.3	0.0 ± 2.1	n.d.

[a]. IC_{50} value is defined as the concentration at 50% inhibition on melanin synthesis, calculated from corresponding dose response curves;

[b]. Inhibition rate (%) = 100% − melanin synthesis (%) of each tested compound;

[c]. n.d.: not determined;

[d]. Arbutin was used as positive control and showed the IC_{50} value of 976.5 μM.

Table S3. Anti-allergy and anti-inflammatory effect of triterpenoids at 25 μg/ml.

No.	Cell viability (%)	Inhibition rate (%)	No.	Cell viability (%)	Inhibition rate (%)
**1**[a]	103.1 ± 5.3	72.5 ± 3.0**	**10**	77.0 ± 5.3**	22.4 ± 1.9
**2**	102.0 ± 6.2	5.8 ± 6.7	**11**	62.6 ± 1.1**	n.d.
**3**	110.2 ± 10.2	29.9 ± 3.3	**12**	93.9 ± 7.3	74.5± 1.3**
**4**	97.9 ± 10.6	29.4 ± 8.1	**13**	85.6 ± 4.2*	n.d
**5**	105.8 ± 1.1	8.5 ± 9.5	**14**	66.8 ±11.1**	n.d.
**6**	97.6 ± 14.1	2.4 ± 14.1	**15**	102.1 ± 2.2	0.9 ± 0.0
**7**	84.4 ± 0.5*	66.0 ± 2.6*	**16**	99.9 ± 2.3	n.d.
**8**	80.4 ± 1.6**	36.1 ± 6.7	**17**	99.8 ± 7.0	n.d.
**9**	98.0 ± 7.7	54.4 ± 4.9*	**18**	104.6 ± 6.7	n.d.
